# Hyperprogressive disease during atezolizumab plus bevacizumab treatment in patients with advanced hepatocellular carcinoma from Japanese real-world practice

**DOI:** 10.1186/s12876-023-02731-5

**Published:** 2023-03-31

**Authors:** Sae Yumita, Sadahisa Ogasawara, Miyuki Nakagawa, Susumu Maruta, Tomomi Okubo, Norio Itokawa, Yotaro Iino, Masamichi Obu, Yuki Haga, Atsuyoshi Seki, Tadayoshi Kogure, Takamasa Ishino, Keita Ogawa, Kisako Fujiwara, Terunao Iwanaga, Naoto Fujita, Takafumi Sakuma, Ryuta Kojima, Hiroaki Kanzaki, Keisuke Koroki, Masanori Inoue, Kazufumi Kobayashi, Soichiro Kiyono, Masato Nakamura, Naoya Kanogawa, Tomoko Saito, Takayuki Kondo, Ryo Nakagawa, Shingo Nakamoto, Ryosuke Muroyama, Tetsuhiro Chiba, Ei Itobayashi, Masanori Atsukawa, Yoshihiro Koma, Ryosaku Azemoto, Kenji Ito, Hideaki Mizumoto, Jun Kato, Naoya Kato

**Affiliations:** 1 Department of Gastroenterology, Graduate School of Medicine, Chiba University, 1-8-1 Inohana, Chuo-Ku, Chiba, Japan; 2 Department of Gastroenterology, Asahi General Hospital, Asahi, Japan; 3 Department of Gastroenterology, Nippon Medical School Chiba Hokusoh Hospital, Inzai, Japan; 4 Department of Internal Medicine, Division of Gastroenterology and Hepatology, Nippon Medical School, Tokyo, Japan; 5 Department of Gastroenterology, Kimitsu Chuo Hospital, Kisarazu, Japan; 6 Department of Gastroenterology, National Hospital Organization Chiba Medical Center, Chiba, Japan; 7 Department of Gastroenterology, Funabashi Municipal Medical Center, Funabashi, Japan

**Keywords:** Hyperprogressive disease, Atezolizumab and bevacizumab, Hepatocellular carcinoma, Immunotherapy

## Abstract

**Background:**

Hyperprogressive disease (HPD) is a phenomenon with greatly accelerated tumor growth and clinical deterioration rates compared to pre-therapy, in patients treated with immune checkpoint inhibitors (ICI). The aim of this study is to clarify the reality of HPD in patients with advanced hepatocellular carcinoma (HCC) who were treated with atezolizumab plus bevacizumab (Atez/Bev) using tumor dynamics.

**Methods:**

Medical records of consecutive patients with advanced HCC who were treated with Atez/Bev were retrospectively reviewed. HPD was defined as a more than two- or fourfold increase in tumor growth rate (TGR) or tumor growth kinetics rate (TGK_R_) before and after treatment. Overall survival (OS) and baseline characteristics with or without HPD were analyzed.

**Results:**

A total of 85 patients were included in the analysis. When HPD was defined as a twofold of TGR or TGK_R_, 8 patients (8/85, 9.4%) had HPD and 11 had PD without HPD. A total of 5 patients (5/85, 5.9%) were diagnosed with HPD and 14 with PD without HPD when HPD was defined as a fourfold of TGR or TGK_R_. No significant difference was observed in the baseline characteristics between HPD and non-HPD.

**Conclusion:**

The prevalence of HPD in patients with advanced HCC treated with Atez/Bev was lower than those treated with nivolumab monotherapy. The HPD mechanism in ICI combined with antibodies targeting vascular endothelial growth factor (VEGF) remains to be elucidated.

**Supplementary Information:**

The online version contains supplementary material available at 10.1186/s12876-023-02731-5.

## Introduction

Hepatocellular carcinoma (HCC) is ranked as the 7th most common neoplasm and the second leading cause of cancer-related death worldwide [1]. Although majority of worldwide guidelines have suggested screening of high-risk population with viral hepatitis carriers or/and cirrhosis [2], many patients are still diagnosed after reaching advanced HCC. Therefore, systemic therapies are critical to improve the prognosis of patients with HCC [3]. Immunotherapy for advanced HCC started to occur somewhat later than that for other carcinomas. Although anti-programmed cell death-1 (PD-1) antibody monotherapies (nivolumab and pembrolizumab) demonstrated antitumor activities in phase II and randomized III trials [4–7], none of them could show survival benefits in the randomized phase III trial. At 2020, the combination of atezolizumab (antibodies targeting programmed cell death ligand 1 [PD-L1]) plus bevacizumab (antibodies targeting vascular endothelial growth factor [VEGF]) (Atez/Bev) was shown in global phase III trial (IMbrave 150) to significantly prolong overall survival (OS) compared to sorafenib alone in the treatment of patients with advanced HCC who did not have any previous history of systemic therapy. Based on this result, the combination immunotherapy has been the standard first-line systemic therapy for advanced HCC [8]. This change has made immunotherapy the mainstay of systemic therapy in advanced HCC as well as other cancers [9–12].

Immune checkpoint inhibitor (ICI) therapy has revealed and noted a unique tumor progression called hyperprogressive disease (HPD) when used for the management of various malignancies in clinical practice. HPD is a phenomenon with greatly accelerated tumor growth and clinical deterioration rates compared to pre-therapy [13, 14], has reported an incidence of 9–29% in various cancer types (mixed solid tumors, non-small cell lung cancers, advanced gastric cancer, and head and neck cancers) [15–17]. Several reports demonstrated that HPD was a poor prognostic factor due to extremely rapid tumor growth. However, HPD has various definitions in previous reports. In other words, there is no fixed definition of HPD at this time, and thus, its definition should be unified to evaluate HPD and to clarify the reality of HPD. The mechanisms that cause HPD in malignancies other than HCC are progressively being understood [18]. Kamada et al*.* recently reported that ICI might promote the proliferation of highly suppressive PD-1 + eTreg cells in HPDs, resulting in the inhibition of antitumor immunity. This study examined patients of advanced stomach cancer where tumor biopsy samples were reasonably straightforward to collect. The mechanism of HPD in HCC, when tumor specimens are challenging to collect, is still unknown.

Kim et al*.* [19] recently reported that 12.7% of patients with advanced HCC receiving nivolumab monotherapy had HPD. Although nivolumab was given to all patients as a second-line treatment after sorafenib, this article was the first to document the incidence rate of HPD in advanced HCC patients receiving ICI. Another report from Japan initially demonstrated the occurrence rate of HPD in patients with advanced HCC who got Atez/Bev in either the first or later lines [20]. According to the article, advanced HCC patients who got Atez/Bev had a 10.2% HPD rate. Although both reports identified patients with HPD with tumor growth dynamics such as tumor growth rate (TGR) and tumor growth kinetics ratio (TGK_R_), the definition for increasing rate differed in each report. Namely, at present, whether HPD differs between ICI monotherapy and combination immunotherapy in patients with advanced HCC remains unclear. Therefore, this study aimed to clarify the prevalence of HPD in patients treated with Atez/Bev based on several examinations using tumor dynamics.

## Methods

### Patients

We retrospectively collected data of consecutive patients with advanced HCC who received Atez/Bev in six institutions in Japan between October 2020 and July 2021. In this analysis, the observation period lasted until the end of October 2021. Patients who did not undergo appropriate radiological assessments that can be evaluated as described in radiological evaluation were excluded. Those with pseudoprogression were also excluded based on the definition that was evaluated as initial progression followed by complete response (CR) or partial response (PR) or stable disease (SD) lasting > 6 months by RECIST v1.1 [21, 22].

Following data were obtained at the first dose of Atez/Bev: sex, age, hepatitis B virus (HBV) positive, hepatitis C virus (HCV) positive, Child–Pugh score, alpha-fetoprotein (AFP) value, Barcelona Clinic Liver Cancer (BCLC) stage, macrovascular invasion, extrahepatic spread, treatment line of Atez/Bev, Eastern Cooperative Oncology Group performance status (ECOG-PS), and neutrophil-to-lymphocyte ratio (NLR). This study was approved by the Research Ethics Committee of the Graduate School of Medicine, Chiba University (No. 3091).

### Radiological evaluation

Patients who underwent three successive computed tomography (CT) or magnetic resonance imaging (MRI) scans available for a RECIST v1.1 assessment: a baseline scan before the first administration of Atez/Bev, a first response assessment (experimental) scan within 12 weeks, and a reference scan within 2 weeks to 3 months before the baseline scan. The reference period was defined as the time between reference and baseline scans, whereas the experimental period was defined as the time between the baseline and experimental scans.

### Definitions of tumor growth dynamics and HPD

Tumor growth dynamics were evaluated based on both TGR and TGK_R_, based on the definitions of previous studies [13, 17, 23]. Target lesions included all measurable lesions up to a maximum of two lesions per organ and a total of five lesions selected from the largest lesions according to RECIST v1.1. HPD was evaluated for both more than two- or fourfold increase in TGR or TGK_R_ of the experimental period compared with that of the reference period in patients with progressive disease (PD) by RECIST v1.1 at the first response evaluation after Atez/Bev administration, respectively. To provide a more objective assessment, at least two physicians retrospectively evaluated the radiological findings: the doctor in charge at each institution and the doctor from Chiba University Hospital, a high-volume center for advanced HCC (S.Y.). At Chiba University Hospital, the radiological evaluations were reviewed by two or more doctors (including S.Y.).

### Statistical analysis

The Pearson’s χ^2^ test or Fisher’s exact test, as appropriate was used to compare clinical characteristics. Data for continuous variables were also categorically evaluated in this study after being split into two groups using the proper cutoffs (age, AFP value, NLR). Based on the receiver operating characteristic curve analysis, the cutoff value for NLR, which was derived as the ratio of the neutrophil count to the lymphocyte count, was established. Kaplan–Meier plots of medians with 95% confidence intervals (CIs) were used to estimate the OS. The final follow-up date was used to determine the censoring date. To compare the OS between the PD without HPD and PD with HPD groups, the log-rank test was used (two-group comparison). With the progression date determined by RECIST v1.1 and the censoring date determined to be the date of the last radiological assessment without progression, the progression-free survival (PFS) following Atez/Bev was calculated using Kaplan–Meier plots of medians with 95% CIs. The time from the date of the initial administration of Atez/Bev and the information from the last observation or death was referred to as the median observation period. Univariate and multivariate logistic regression model were performed to identify predictive factors associated with HPD. The variables for this analysis were chosen from two prior studies of HPD in advanced HCC patients treated with ICIs [19, 20] as well as from general parameters that affect the prognosis of advanced HCC, such as liver function, tumor factors, and tumor markers. All *P*-values < 0.05 were considered statistically significant. All statistical analyses were performed using Statistical Package for the Social Sciences statistical software version 25 (IBM, Chicago, IL, USA).

## Results

### Patient characteristics

During the study period, 147 patients with advanced HCC received Atez/Bev at six Japanese institutions. Among these 147 patients, 59 who did not undergo appropriate radiological examinations (CT or MRI) at pre- or post-treatment and 3 who were diagnosed with pseudoprogression were excluded. Finally, a total of 85 patients were included in the analysis.

The baseline characteristics of these 85 patients are summarized in Table 1. The majority of patients (73/85, 85.9%) were male, and the median age was 74 (range: 48–88) years. During the Atez/Bev administration, most of the patients were classified as Child–Pugh scores of 5 or 6 (79/85, 92.9%) and ECOG-PS of 0 or 1 (85/85, 100%). In the current cohort, 46 patients (54.1%) received Atez/Bev as a second or later line of therapy following other systemic therapy. The most common first-line treatment of patients who administrated Atez/Bev as second or later were lenvatinib (35/46, 76.1%), sorafenib (8/46, 17.4%), and investigational treatment with ICIs (3/46, 6.5%). As the disease progressed on radiological imaging, the majority of patients who had previously had systemic treatment for Atez/Bev stopped taking it (40/46, 87.0%).

**Table 1:** Baseline characteristics of 85 patients with advanced hepatocellular carcinoma who received atezolizumab plus bevacizumab

**Characteristic**	***N* = 85**
Sex, male, *n* (%)	73 (85.9)
Age > 74, *n* (%)	41 (48.2)
HBV positive, *n* (%)	18 (21.2)
HCV positive, *n* (%)	25 (29.4)
Child–Pugh class A, *n* (%)	79 (92.9)
AFP > 400 ng/mL, *n* (%)	32 (37.6)
BCLC stage C, n (%)	52 (61.2)
Macrovascular invasion, *n* (%)	18 (21.2)
Extrahepatic spread, *n* (%)	35 (41.2)
Treatment line, atezolizumab plus bevacizumab,1st line, *n* (%)	39 (45.9)
ECOG-PS ≤ 1, *n* (%)	85 (100)
NLR ≥ 3.43, *n* (%)	35 (41.2)

*Abbreviations*: *HBV* hepatitis B virus, *HCV* hepatitis C virus, *AFP* alpha-fetoprotein, *BCLC* Barcelona Clinic Liver Cancer, *ECOG-PS* Eastern Cooperative Oncology Group Performance Status, *NLR* neutrophil-to-lymphocyte ratio

Each of the 39 patients who received Atez/Bev as the first-line of treatment during the reference period did not get any treatment. In contrast, 80.4% (37/46) of the 46 patients who received Atez/Bev in the second or later line had previously received treatment during the reference period. The reference period's median length in these individuals was 1.4 (within a range of 0.5–3.0) months, and the treatment's median length was 1.1 (within a range of 0.1–2.5) months.

In the present cohort, the median observation period was 6.2 (range: 1.1–12.2) months, and the median number of radiological assessments was 2 (range: 1–8) times. During the observation period, 57 patients discontinued Atez/Bev and 28 patients transitioned to post-treatment after Atez/Bev. Lenvatinib (9 patients), cabozantinib (9 patietns), and sorafenib (3 patients) were the most often prescribed post-Atez/Bev treatments, respectively. The current cohort's median progression-free survival (PFS) and overall survival (OS) were 5.5 months (95% CI, 4.3–6.8) and undefined, respectively (Fig. S1 A and B). These had survival rates of 80.6% at six months and 51.7% at twelve months, respectively.

### Treatment outcomes and identification of HPD in patients treated with Atez/Bev in the present cohort

At the initial radiological evaluation, CR, PR, SD, and PD were observed in 1 (1.2%), 4 (4.7%), 61 (71.8%), 19 (22.4%) patients, respectively. The best radiological responses for CR, PR, SD, and PD during the observation period were 1 (1.2%), 9 (10.6%), 58 (68.2%), and 19 (22.4%). Six of the 19 individuals who were determined to have PD underwent radiological evaluations twice or more to confirm disease progression. We determined PD in the remaining 13 patients with a single radiological assessment that took into account many clinical characteristics (elevation of AFP value: 5 patients, deterioration of ECOG-PS: 8 patients, and deterioration of Child–Pugh score: 4 patients).

**Fig. 1:**
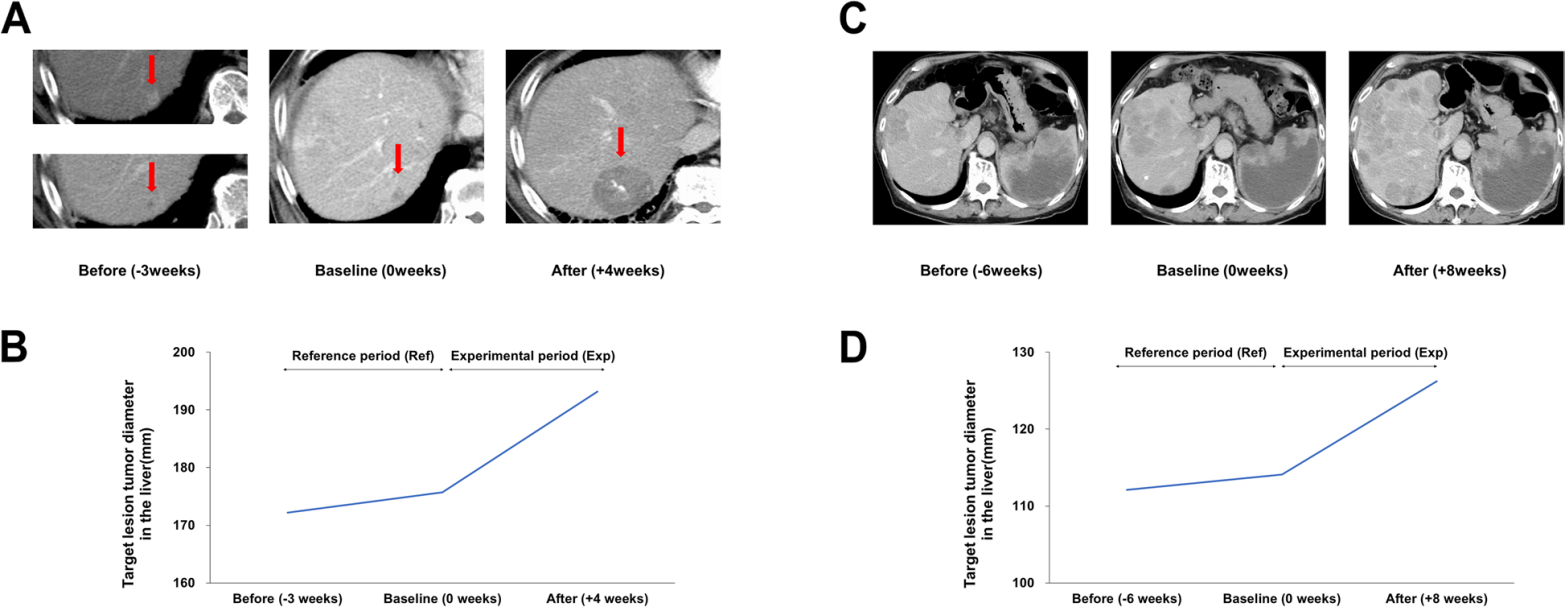
Representative cases of HPD (defined as a fourfold of TGR or TGK_R_) in patients with advanced HCC treated Atez/Bev. **A** and **B** Changes in CT scans and target lesions tumor diameter in the liver of the case who received Atez/Bev as the first-line treatment. **C** and **D** Changes in CT scans and target lesion tumor diameter in the liver of the case who received Atez/Bev as the second-line treatment. HPD indicates hyperprogressive disease

To evaluate the HPD incidence, the tumor growth dynamics were assessed using TGR and TGK_R_ [13, 17, 23]. When HPD was identified using the HPD definition according to a twofold or more increase in TGR or TGK_R_, 8 (8/85, 9.4%) and 11 patients were found to have HPD and PD without HPD, respectively. Conversely, 5 (5/85, 5.9%) patients were identified to have HPD and 14 patients had PD without HPD when HPD was defined as a fourfold or more increase in TGR or TGK_R._ Two typical cases of HPD in patients with advanced HCC are shown in Fig. 1 based on the fourfold or more increase in TGR or TGK_R_ (left column, first-line patient; right column, second-line patient).

The comparison of baseline characteristics in patients with and without HPD is summarized in Table 2. No significant difference was observed in the baseline characteristics between patients with and without HPD using either definition in the present cohort. Additionaly, univariate and multivariate analysis were performed with logistic regression analysis of factors associated with HPD, but there was no significant difference in the baseline characteristics (Table S1 and S2).

**Table 2:** Comparison of baseline characteristics in patients with advanced hepatocellular carcinoma with and without hyper-progressive disease according to two different criteria

**Characteristic**	**TGR ≥ 2 or TGK**_**R**_** ≥ 2**			**TGR ≥ 4 or TGK**_**R**_** ≥ 4**		
	**HPD** ***N* = 8**	**Non-HPD** ***N* = 77**	***P*-value**	**HPD** ***N* = 5**	**Non-HPD** ***N* = 80**	***P*-value**
Sex, male, *n* (%)	7 (87.5)	66 (85.7)	1.000	5 (100)	68 (85.0)	1.000
Age > 74 years, *n* (%)	3 (37.5)	38 (49.4)	0.714	3 (60.0)	38 (47.5)	0.669
HBV positive, *n* (%)	1 (12.5)	17 (22.1)	1.000	0 (0.0)	18 (22.5)	0.579
HCV positive, *n* (%)	4 (50.0)	21 (27.3)	0.226	3 (60.0)	22 (27.5)	0.149
Child–Pugh class A, *n* (%)	7 (87.5)	72 (93.5)	0.458	5 (100)	74 (92.5)	1.000
AFP > 400 ng/mL, *n* (%)	3 (37.5)	29 (37.7)	1.000	2 (40.0)	30 (37.5)	1.000
BCLC stage C, n (%)	5 (62.5)	47 (61.0)	0.257	3 (60.0)	49 (61.3)	1.000
Macrovascular invasion, *n* (%)	1 (12.5)	17 (22.1)	1.000	0 (0.0)	18 (22.5)	0.579
Extrahepatic spread, *n* (%)	2 (25.0)	33 (42.9)	0.461	1 (20.0)	34 (42.5)	0.645
Treatment line, 1st line, *n* (%)	2 (25.0)	37 (48.1)	0.279	2 (40.0)	37 (46.3)	1.000
ECOG-PS ≤ 1, *n* (%)	8 (100)	77 (100)	1.000	5 (100)	80 (100)	1.000
NLR ≥ 3.43, *n* (%)	5 (62.5)	30 (39.0)	2.464	4 (80.0)	31 (38.8)	0.154

*Abbreviations*: *HBV* hepatitis B virus, *HCV* hepatitis C virus, *AFP* alpha-fetoprotein, *BCLC* Barcelona Clinic Liver Cancer, *ECOG-PS* Eastern Cooperative Oncology Group Performance Status, *NLR* neutrophil-to-lymphocyte ratio

**Fig. 2:**
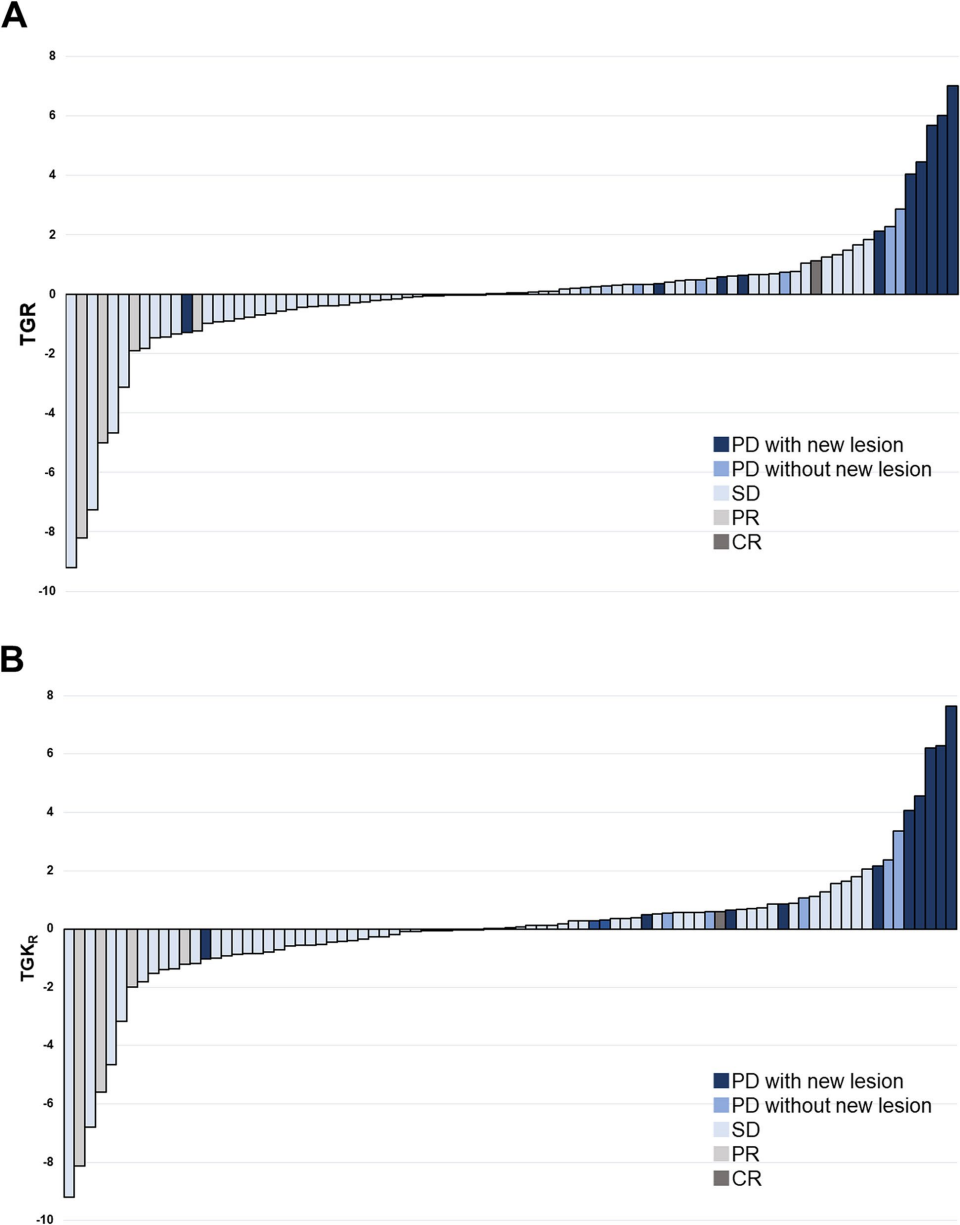
**A** TGR (experimental TGR/reference TGR) and (**B**) TGK_R_ (experimental TGK/reference TGK) distribution. HPD indicates hyperprogressive disease; PD, progressive disease (per RECIST v1.1); SD, stable disease (per RECIST v1.1); PR, partial response (per RECIST v1.1); CR, complete response (per RECIST v1.1)

We illustrated TGR and TGK_R_ distributions of all study population in Fig. 2. According to the definition of twofold or more increase in TGR or TGK_R_, the median increase in TGR and TGK_R_ in patients with HPD was 4.25 (range 2.11 to 7.03) and 4.07 (range 2.06 to 7.63), respectively. Conversely, the median increases of TGR and TGK_R_ in patients without HPD were − 0.02 (range − 9.21 to 1.84) and − 0.03 (range − 9.21 to –1.78), respectively. The median increases of TGR and TGK_R_ in patients with HPD were 5.68 (range 4.05 to 7.63) and 6.21 (range 4.07 to 7.63), respectively, based on the definition of fourfold or more increase of TGR or TGK_R_. Likewise, the median increases of TGR and TGK_R_ in patients without HPD were − 0.00 (range − 9.21 to 2.87) and − 0.00 (range − 9.21 to 2.38), respectively. All five patients identified as HPD based on the definition of fourfold or more increase of TGR or TGK_R_ observed new lesions at the same time when deemed to PD.

We performed an additional analysis that excluded patients who received other systemic therapies during the reference period. In the preset cohort, 9 of 46 patients who recived Atez/Bev in the second or later lines did not receive any treatment during the reference period. A total of 48 patients, including these 9 patients and 39 patients who received Atez/Bev in the first line, did not receive any treatment during the reference period. The baseline characteristics of these 48 patients are summarized in Table S3. The incidence of HPD by definitions according to a twofold or more and a fourfold or more increase in TGR or TGK_R_ was 6.3% (3 patinets) and 4.2% (2 patients), respectively. The comparison of baseline characteristics in patients with and without HPD is summarized in Table S4.

### Tumor growth dynamics using TGR and TGKR associations between HPD

**Fig. 3:**
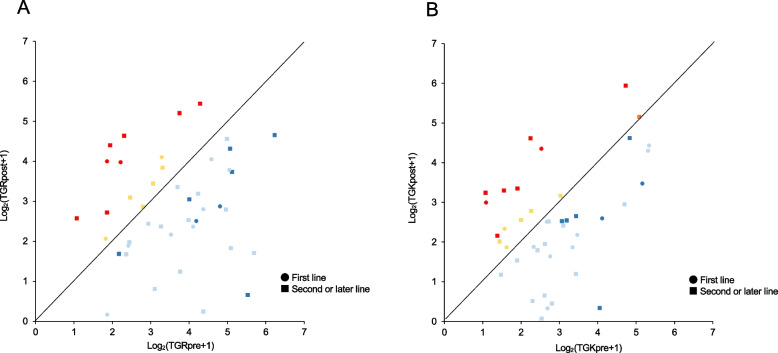
Analysis of tumor growth dynamics between the reference and experimental periods in patients in this cohort. Pairwise comparisons of log-transformed TGR (**a**) and TGK_R_ (**b**) between the reference time and experimental periods. Red color; HPD, Orange color; PD with accelerated TGK_R_ but without HPD, Blue color; PD with decelerated TGR or TGK_R_, Light Blue color; SD with decelerated TGR or TGK_R_

In the preset cohort, we compared the correlation between treatment response and tumor growth dynamics using log-transformed TGR and TGK_R_ based on the reference and experimental periods (Fig. 3A and B). The majority of patients achieved a deceleration in the growth speed of their tumors with Atez/Bev even if the best tumor response was PD according to RECIST v1.1 (blue dots in Fig. 3A and B). Conversely, the majority of accelerated tumor growth was observed in patients treated with Atez/Bev after second- or later-lines (10 of 14 patients in TGR and 11 of 15 patients in TGK_R_).

### Changes of liver function and general status in advanced HCC patients with HPD during Atez/Bev treatment

We evaluated changes in liver function and the general status during Atez/Bev treatment in patients with advanced HCC. Of the 85 patients who were who included this study, 83, and 85 patients, respectively, were able to have their baseline and 12-week post-treatment Child–Pugh scores and ECOG-PS measurements made. In the current study, patients with PD and HPD had a tendency for their Child–Pugh scores and ECOG-PS to deteriorate at week 12 compared to baseline (Table S5).

### Impact of HPD on survival in patients with advanced HCC who received Atez/Bev in the real-world practice

**Fig. 4:**
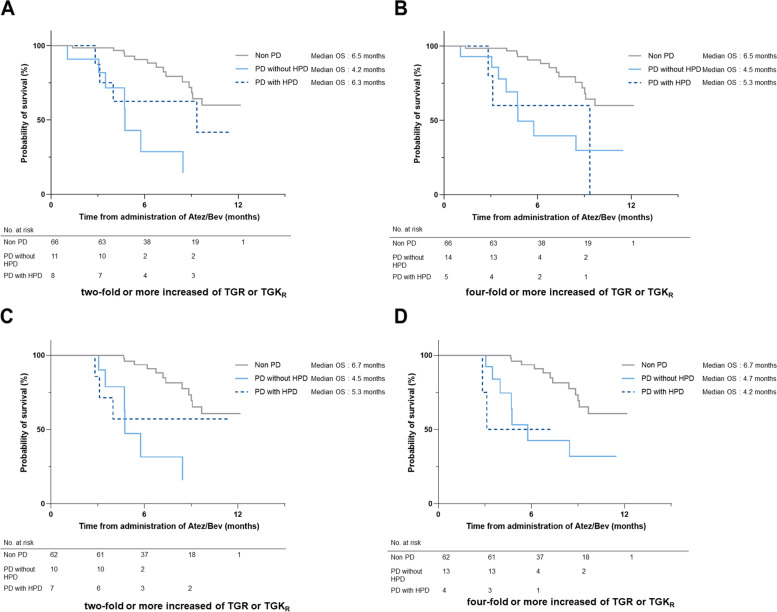
Overall survival of HPD and PD without HPD and non-PD. HPD was defined as a more than twofold (**A**) or fourfold (**B**) increases of TGR or TGK_R_ in patients determined to have PD by RECIST v1.1 at the first response. Landmark survival analyses at 12 weeks to compare OS between PD without HPD, and PD with HPD groups were also analyzed as a more than twofold (**C**) or fourfold (**D**) increases of TGR or TGK_R_ in patients determined to have PD by RECIST v1.1 at the first response

Figure 4A and B shows Kaplan–Meier curves of OS in patients with advanced HCC who received Atez/Bev. We compared OS between non-PD, PD without HPD, and PD with HPD groups in these analyses. In both definitions of two- and fourfold or more increase of TGR or TGK_R_, no significant differences were observed between PD with and without HPD (twofold: *P* = 0.233, fourfold: *P* = 0.969). Additionally, landmark survival analyses at 12 weeks to compare OS between PD without HPD, and PD with HPD groups were analyzed in both definitions (Fig. 4C and D). These results showed no significant difference between PD with and without HPD (twofold: *P* = 0.513, fourfold: *P* = 0.721).

Recently, a grading system known as the CRAFITY score was established to forecast how patients with advanced HCC who receive ICI will fare [24]. We could evaluate 84 of 85 patients with advanced HCC treated by Atez/Bev in the present cohort. Fig. S2 reveals Kaplan–Meier curves of OS according to the CRAFITY scores. There was significant stratification of prognosis by in the CRAFITY score (*P* = 0.006). Table S6 provides the CRAFITY scores for the non-PD, PD without HPD, and PD with HPD groups. We observed no significant difference in the CRAFITY scores among groups of non-PD, PD without HPD, and PD with HPD according to both definitions of two- and fourfold or more increase of TGR or TGK_R_.

## Discussion

This study clarified the reality of HPD in patients with advanced HCC treated with Atez/Bev using two different definitions based on tumor dynamics immediately before and after Atez/Bev administration. In particular, HPD in Atez/Bev was confirmed to be lesser than ICI monotherapy in patients with advanced HCC, as evaluated based on the definition set out in the previous report [19]. Unfortunately, this study could not identify any clinical parameter that predicted HPD in patients with advanced HCC treated with Atez/Bev.

In the present study, HPD was evaluated based on the definition of two- and fourfold or greater increase of TGR or TGK_R_ with reference on a previous study [19]. Several current studies that analyzed HPD in other cancers adopted the definition of twofold or greater increase of TGR or TGK_R_ [15–17]. The majority of reports on other cancers using this definition were analyses of cohorts including ICI as the first-line treatment. Conversely, Kim et al*.* recently reported that the HPD frequency in ICI monotherapy as second- or later-lines treatment for advanced HCC used the definition of fourfold or greater increase of TGR or TGK_R_ [19]. The definition here was established based on the findings that no patient with advanced HCC had increased tumor growth dynamics using TGR and TGK_R_ higher than fourfold who converted from sorafenib as first-line treatment to other ICIs, such as regorafenib or best-supportive care. The presence or absence of pre-treatment should have different implications for tumor growth dynamics using TGR and TGK_R_, as determined by changes in the tumor growth rate between the period immediately before and after initiating ICI treatment. This is because the tumor growth in the period immediately before ICI treatment is influenced by the previous systemic therapy in the analysis of tumor growth dynamics using TGR and TGK_R_ in patients who received ICI as second- or later-lines treatment. In other words, tumor growth acceleration after ICI administration as second- or later-lines treatments should be considered not only due to HPD but also due to the lack of efficacy of the previous treatment. Since this study included patients with advanced HCC who started Atez/Bev as both first- and second- or later-lines, we assessed HPD using two different definitions.

We found that the frequency of HPD in patients with advanced HCC treated with Atez/Bev was 5.9% (first-line: 5.1%; second- or later-line: 6.5%) using the same method in the current report by Kim et al*.* [19]*.* This HPD rate was lower than the 12.7% indicated in the study that observed the frequency of HPD in ICI monotherapy as second- or later-lines treatment for patients with advanced HCC. A very recent report from Japan evaluating HPD based the definition of a twofold or greater increase of TGR or TGK_R_ indicated the frequency of HPD as 10.2% [20], which was identical to 9.4% of our results adopting the same definition. Taken together with these results, HPD was likely reduced by anti-VEGF antibody combined with ICI in patients with advanced HCC. ICI combined with anti-VEGF antibody is known to have synergic effects due to the action of an anti-VEGF antibody on tumor microenvironment, including enhancing T-cell priming and activation via promotion of dendritic cell maturation, increasing T-cell tumor infiltration by normalizing tumor vasculature, and establishing an immune-permissive tumor microenvironment by decreasing myeloid-derived suppressor-cell and regulatory T-cell populations [25, 26].

These effects of anti-VEGF antibody toward tumor microenvironment would most likely reduce HPD in patients with advanced HCC treated with Atez/Bev. Several reports have demonstrated the HPD mechanisms in other malignancies [27]. However, most of them were HPD analyses with ICI monotherapy or combination therapy of two different ICIs. To the best of our knowledge, the detailed HPD mechanism during combination treatment of ICI and anti-VEGF antibody has not yet been elucidated.

In the present study, no predictive factors of HPD were identified based on clinical parameters of baseline data at the start of Atez/Bev in patients with advanced HCC. Moreover, a significant difference in OS was not observed between patients with PD with and without HPD in our cohort. Several previous reports demonstrated that NLR was helpful in identifying HPD in both patients with advanced HCC and other cancers [28, 29]. Additionally, the planned study had a number of shortcomings. First, this was a retrospective study based on actual practice. Thus, the timing of the radiological assessment was not strictly defined. Additionally, patients classified as PD in the current study included individuals who had only received a single radiological evaluation by taking into account the clinical progression of other criteria such as tumor markers and general condition. Clinical challenges still exist in the real-world setting when it comes to separating “genuine PD,” including HPD, from pseudoprogression in patients receiving ICI or its combo therapy for advanced HCC. To confirm PD, two or more radiological examinations should be carried out in accordance with irRECIST to differentiate between “genuine PD,” including HPD, and pseudoprogression. Second, the observation period of this study might be not long enough to assess the impact of HPD due to Atez/Bev on OS. Third, only a limited number of patients in this cohort were identified as HPD during treatment for Atez/Bev (twofold: 8 patients, fourfold: 5 patients). Fourth, both patients who received Atez/Bev in the first and second or later lines were included in our cohort. Additionally, several patients in the second or third line got pre-treatment throughout the reference period. Although we conducted our analysis using the definition of HPD in previous treated advanced HCC patients used in the study by Kim et al*.* the results of this study might be affected by the including patients with diverse treatment histories before Atez/Bev. To learn more about HPD in patients with advanced HCC treated with ICIs, more research in large cohort studies is necessary.

In conclusion, our study confirmed that the prevalence of HPD in patients with advanced HCC treated with Atez/Bev was lower than that of nivolumab monotherapy. Although the combination of anti-VEGF antibody in ICI suppressed to occurrence of HPD and negative clinical impact of HPD was reduced in patients with advanced HCC, elucidation of the HPD mechanism in ICI combined with anti-VEGF antibody and identification of its predictors are strongly required in patients with advanced HCC.

## Supplementary Information

**Additional file 1: Supplementary Fig. 1.** Progression-free survival (A) and overall survival (B) of the whole study population. **Supplementary Fig. 2.** Overall survival according to the CRAFITY scores. **Supplementary Table 1.** Univariate and multivariate Logistic regression analyses of factors associated with HPD (defined as a twofold of TGR or TGK_R_). **Supplementary Table 2.** Univariate and multivariate logistic regression analyses of factors associated with HPD (defined as a fourfold of TGR or TGK_R_). **Supplementary Table 3.** Baseline characteristics of 48 patients with advanced hepatocellular carcinoma who received atezolizumab plus bevacizumab with no therapeutic intervention in the reference period. **Supplementary Table 4.** Comparison of baseline characteristics in patients with advanced hepatocellular carcinoma who received atezolizumab plus bevacizumab with no therapeutic intervention in the reference period. **Supplementary Table 5.** Changings of Child–Pugh score and ECOG-PS during at baseline and at 12 weeks after starting treatment. **Supplementary Table 6.** CRAFTY scores for each of non-PD, PD without HPD, and PD with HPD groups.

## Data Availability

Datasets generated and analyzed during the current study are available from the corresponding author on reasonable request.
