# Supplementary material for: Hyperprogressive disease during atezolizumab plus bevacizumab treatment in patients with advanced hepatocellular carcinoma from Japanese real-world practice
Source: BMC Gastroenterol. 2023 Mar 31;23:101. doi: 10.1186/s12876-023-02731-5 (PMC10067175; doi:10.1186/s12876-023-02731-5)
Supplement: Supplementary file 1 — Additional file 1: Supplementary Fig. 1. Progression-free survival (A) and overall survival (B) of the whole study population. Supplementary Fig. 2. Overall survival according to the CRAFITY scores. Supplementary Table 1. Univariate and multivariate Logistic regression analyses of factors associated with HPD (defined as a twofold of TGR or TGKR). Supplementary Table 2. Univariate and multivariate logistic regression analyses of factors associated with HPD (defined as a fourfold of TGR or TGKR). Supplementary Table 3. Baseline characteristics of 48 patients with advanced hepatocellular carcinoma who received atezolizumab plus bevacizumab with no therapeutic intervention in the reference period. Supplementary Table 4. Comparison of baseline characteristics in patients with advanced hepatocellular carcinoma who received atezolizumab plus bevacizumab with no therapeutic intervention in the reference period. Supplementary Table 5. Changings of Child–Pugh score and ECOG-PS during at baseline and at 12 weeks after starting treatment. Supplementary Table 6. CRAFTY scores for each of non-PD, PD without HPD, and PD with HPD groups. [file 12876_2023_2731_MOESM1_ESM.zip › Supl_Table 3,4.docx]

**Supplementary Table 3.** Baseline characteristics of 48 patients with advanced hepatocellular carcinoma who received atezolizumab plus bevacizumab with no therapeutic intervention in the reference period.

| **Characteristic** | **N = 48** |
| --- | --- |
| Sex, male, *n* (%) | 40 (83.3) |
| Age > 74, *n* (%) | 26 (54.2) |
| HBV positive, *n* (%) | 07 (14.6) |
| HCV positive, *n* (%) | 14 (29.2) |
| Child–Pugh class A, *n* (%) | 46 (95.8) |
| AFP > 400 ng/mL, *n* (%) | 16 (33.3) |
| BCLC stage C, n (%) | 24 (50.0) |
| Macrovascular invasion, *n* (%) | 10 (20.8) |
| Extrahepatic spread, *n* (%) | 13 (27.1) |
| Treatment line, atezolizumab plus bevacizumab,1st line, *n* (%) | 39 (81.3) |
| ECOG-PS ≤ 1, *n* (%) | 48 (100) |
| NLR ≥ 3.43, *n* (%) | 14 (29.2) |

Abbreviations: HBV, hepatitis B virus; HCV, hepatitis C virus; AFP, alpha-fetoprotein; BCLC, Barcelona Clinic Liver Cancer; ECOG-PS, Eastern Cooperative Oncology Group Performance Status; NLR, neutrophil-to-lymphocyte ratio.

**Supplementary Table 4.** Comparison of baseline characteristics in patients with advanced hepatocellular carcinoma who received atezolizumab plus bevacizumab with no therapeutic intervention in the reference period.

| **Characteristic** | **TGR ≥ 2 or TGK_R_ ≥ 2** | | | **TGR ≥ 4 or TGK_R_ ≥ 4** | | |
| --- | --- | --- | --- | --- | --- | --- |
|  | **HPD**  **N = 3** | **Non-HPD**  **N = 45** | ***P*-value** | **HPD**  **N = 2** | **Non-HPD**  **N = 46** | ***P*-value** |
| Sex, male, *n* (%) | 3 (100) | 37 (82.2) | 1.000 | 2 (100) | 38 (82.6) | 1.000 |
| Age > 74 years, *n* (%) | 2 (66.7) | 24 (53.3) | 1.000 | 2 (100) | 24 (52.2) | 0.493 |
| HBV positive, *n* (%) | 0 (0.0) | 7 (15.6) | 1.000 | 0 (0.0) | 7 (15.2) | 1.000 |
| HCV positive, *n* (%) | 0 (0.0) | 14 (31.1) | 0.546 | 0 (0.0) | 14 (30.4) | 1.000 |
| Child–Pugh class A, *n* (%) | 2 (66.7) | 44 (97.8) | 0.122 | 1 (50.0) | 45 (97.8) | 0.082 |
| AFP > 400 ng/mL, *n* (%) | 1 (33.3) | 15 (33.3) | 1.000 | 1 (50.0) | 15 (32.6) | 1.000 |
| BCLC stage C, n (%) | 1 (33.3) | 23 (51.1) | 1.000 | 3 (60.0) | 49 (61.3) | 0.489 |
| Macrovascular invasion, *n* (%) | 1 (33.3) | 9 (20.0) | 0.512 | 0 (0.0) | 10 (21.7) | 1.000 |
| Extrahepatic spread, *n* (%) | 0 (0.0) | 13 (28.9) | 0.553 | 0 (0.0) | 13 (28.3) | 1.000 |
| Treatment line, 1st line, *n* (%) | 2 (66.7) | 37 (82.2) | 0.472 | 2 (100.0) | 37 (80.4) | 1.000 |
| ECOG-PS ≤ 1, *n* (%) | 3 (100) | 45 (100) | 1.000 | 2 (100) | 46 (100) | 1.000 |
| NLR ≥ 3.43, *n* (%) | 2 (66.7) | 12 (26.7) | 0.20 | 2 (100) | 12 (26.1) | 0.081 |

Abbreviations: HBV, hepatitis B virus; HCV, hepatitis C virus; AFP, alpha-fetoprotein; BCLC, Barcelona Clinic Liver Cancer; ECOG-PS, Eastern Cooperative Oncology Group Performance Status; NLR, neutrophil-to-lymphocyte ratio.
